# Between‐species contagion drives HPAI transmission and mass mortality in seabirds

**DOI:** 10.1111/1365-2656.70305

**Published:** 2026-07-06

**Authors:** Jason Matthiopoulos, Stephen Votier, Sarah Wanless, Emma Cunningham, Jude V. Lane, Jana W. E. Jeglinski

**Affiliations:** ^1^ School of Biodiversity One Health and Veterinary Medicine University of Glasgow Glasgow UK; ^2^ Lyell Centre Heriot Watt University Edinburgh UK; ^3^ UK Centre for Ecology & Hydrology, Bush Estate Penicuik UK; ^4^ School of Biological Sciences, Ashworth Labs University of Edinburgh Edinburgh UK; ^5^ RSPB Centre for Conservation Science Sandy Bedfordshire UK; ^6^ Department of Ecoscience Aarhus University Roskilde Denmark

**Keywords:** avian flu, black eyes, epidemiological connectivity, H5N1 clade 2.3.4.4b HPAIV, integrated state‐space model, latent processes, network dynamics, perfect mixing

## Abstract

1. The unprecedented avian panzootic caused by High‐Pathogenicity Avian Influenza (HPAI) caused widespread mortality in seabird populations, but the exact scales and patterns of transmission, basic epidemiological parameters and mortality outcomes are unknown. Mass mortalities combined with potentially detrimental sub‐lethal effects of exposure will have currently unpredictable consequences for population viability of affected wild bird species, many of which are already subjected to acute anthropogenic pressures.
2. Here, we focus on one of the most severely affected seabird species, the Northern gannet (*Morus bassanus*). Using a metapopulation epidemiological framework, fitted to counts of dead gannets in or near gannet breeding colonies across the NorthEast Atlantic, we estimate key epidemiological parameters, quantify spatial patterns of mortality and isolate external transmission routes.
3. Our epidemiological parameter estimates suggest that the 2022 strains of HPAI were highly virulent and transmissible over wide geographical ranges, within the gannet network and beyond. Within gannet colonies, we found evidence that transmission rates may have declined as mortality reduced local breeding density.
4. Inter‐colony spread in the network declined with distance, and epidemiological connectivity had an estimated half‐distance of 5 km. Large‐scale outbreak initiation was best explained by external infection sources, most likely, other seabird species, with an estimated half‐distance of 66 km.
5. Our modelling suggests that these multi‐scale, multispecies processes resulted in 28% (95% CI: 21%–34%) HPAI‐mortality across the Northeast Atlantic gannet breeding metapopulation, in addition to normal levels of annual mortality (5%–10%).
6. Our study demonstrates that if analysed holistically, carcass data can provide epidemiological insights but also act as an early warning system for continent‐wide patterns of spread. For pandemic preparedness, it is essential that data‐integrative approaches such as ours are placed in the mainstream toolbox of planetary health monitoring. By disentangling intra‐ and inter‐specific transmission, our approach highlights the need to integrate colonial species into global avian influenza surveillance and response.

## Graphical abstract

Modelling the unprecedented avian panzootic caused by High‐Pathogenicity Avian Influenza (HPAI) across the Northeast Atlantic gannet breeding metapopulation, we estimated 28% (95% CI: 21%–34%) HPAI‐related mortality. Large‐scale outbreak initiation was best explained by external infection sources, most likely, other seabird species.

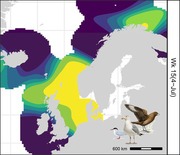

## 1 INTRODUCTION

Wild populations act as the main reservoir of high‐pathogenicity avian influenza (HPAI) (Runstadler & Puryear, 2024) yet, our understanding of HPAI dynamics in wild birds remains limited. Understanding transmission and virulence in wildlife systems is vitally important, for two reasons: First, many affected animal populations are already vulnerable or in decline due to multiple anthropogenic pressures such as habitat loss and direct mortality (Dias et al., 2019; Runstadler, 2024). Additional events of mass mortality and breeding failure brought about by disease can precipitate declines that may be impossible to mitigate via conventional conservation measures. Second, the high mobility of many free‐ranging or migratory animals facilitates the dissemination of HPAI across different environments at both a local and global scale (Yang et al., 2024) and makes them a prime candidate route for spillovers into livestock or humans (Erdelyan et al., 2024).

The recent, rapid emergence of HPAI H5N1 subtype clade 2.3.4.4b in wildlife—at least 26 avian and 17 novel mammalian taxa (Erdelyan et al., 2024; Puryear & Runstadler, 2024)—prompted questions about the detailed mechanisms of contact, contagion and disease persistence within and among species. Despite extensive local reports of high breeding failure and adult mortality across a range of different species (Knief et al., 2024; Lane et al., 2024; McPhail et al., 2025; NatureScot, 2023), thus far we have gained only limited understanding of the spatiotemporal scales and patterns of spread across avian communities.

To assess whether a disease can be sustained in a single‐species system, we must quantify parameters of epidemiological importance such as rates of infection, mortality, recovery and the basic reproductive number ($R_{0}$—the average number of individuals that will contract a contagious disease from one infected individual, in a naive population). However, despite their importance for rapid risk assessment, such empirical parameters are not sufficient for large‐scale prediction. Any kind of spatial structuring, whether at fine or coarse scales, can modify the rate of transmission regionally and propagate small‐scale stochastic events to whole‐system heterogeneities in outbreaks. Such complex, system‐level dynamics can only be captured with the aid of data‐driven modelling. Having accounted for epidemiological patterns in any one focal species, it is then important to know how an outbreak unfolds across the broader, multispecies community, including species for which there may be limited observations.

Analytical approaches based on detailed histology and viral genomics data (Erdelyan et al., 2024; Yang et al., 2024) or high‐frequency observations of long‐range animal movements (Careen et al., 2024; Grémillet et al., 2023; Jeglinski, Lane, et al., 2024) are the scientific gold standard for a deep understanding of disease routes and dynamics; however, our attempts at building insights from first principles can be overwhelmed by the multitude of different mechanisms that need to be investigated and interlinked (see attempt in McPhail et al., 2025). Possible transmission pathways may be intra‐specific via stepping‐stone or large‐scale transitions among sub‐populations or inter‐specific interactions such as scavenging or other mixed‐species close‐contact interactions. In contrast to farmed livestock populations, many wildlife habitats are expansive and inaccessible to direct or even remote observation, not least because access to colonies can be limited by biosecurity and permit issues. Therefore, typical molecular testing and symptomatic approaches are simply not feasible with high enough frequency across continental scales.

Alternatively, we can eschew detailed mechanisms in favour of broad patterns of connectivity and epidemiology inferred from large‐scale mortality data giving clues about disease prevalence and local outbreak timing. Since few wildlife systems have established surveillance networks that can span the range of highly mobile species, the challenge with this approach is that data will necessarily be opportunistic and unbalanced.

The distortive effects of patchy surveillance networks and observation effort heterogeneities are aggravated by variations in the symptomatic appearance and mortality across species (Greco et al., 2025) which mean that mortalities reported for most gravely affected species are not necessarily indicative of the burden in reservoir species. Hence, currently, field studies are inconclusive about the existence of robust spatiotemporal patterns in viral spread. Some studies (Andrew et al., 2024) have not found spatial clustering, even with the benefits of sequencing analyses, whereas others (McPhail et al., 2025) have been able to describe the progression of spatial waves of prevalence. Yet, the fragmentary and highly stochastic reporting of cases should not prevent us from seeking underlying patterns and structure in incidence/prevalence data. It is only through capturing such patterns, that we can begin the process of prediction, in support of prevention.

**FIGURE 1:**
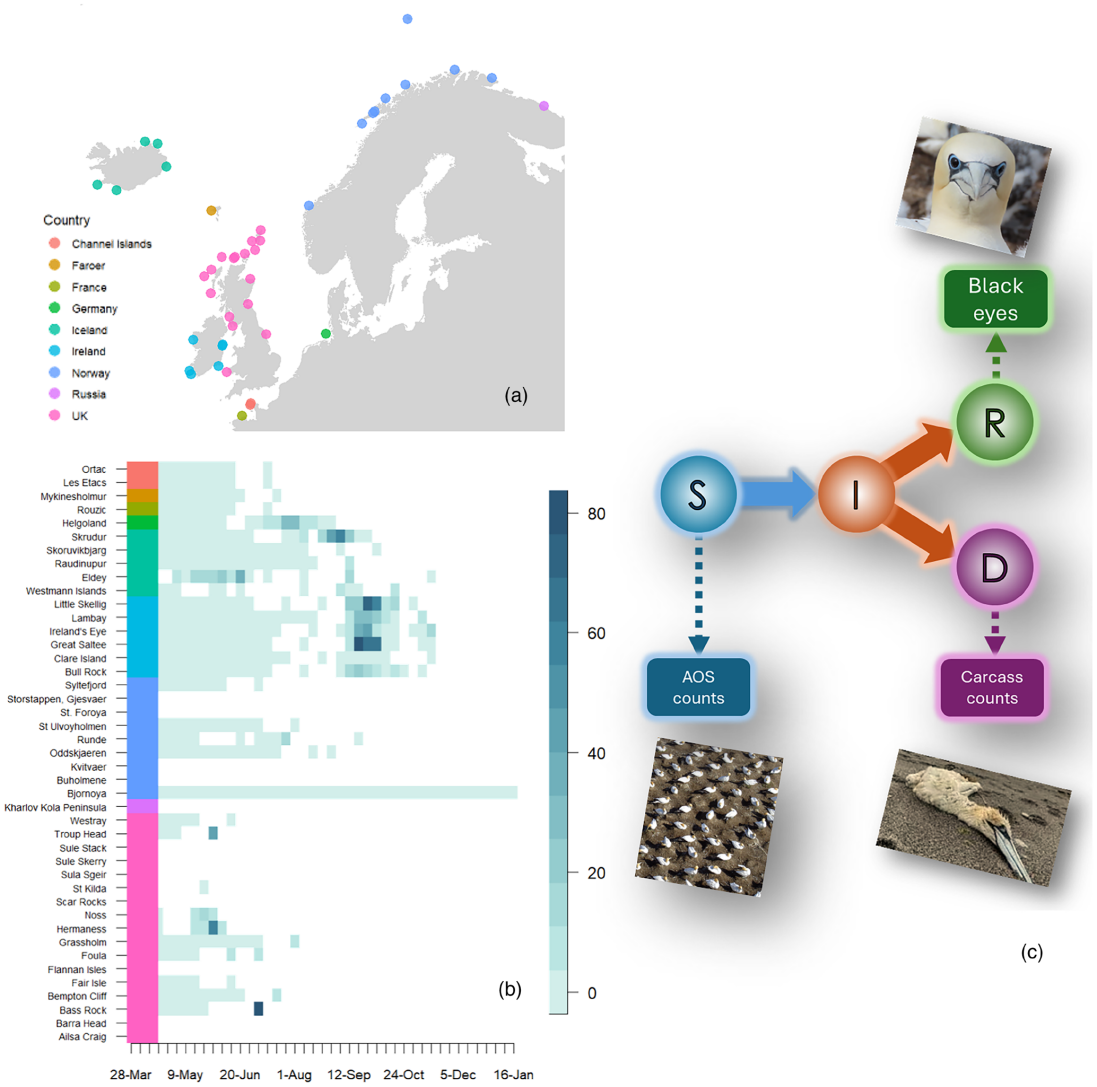
Data overview. (a) Carcass count data were available for 43 gannet breeding colonies, here coloured by country. (b) The availability of carcass data (colour scale curtailed at >80 carcasses, lack of data shown in white) varied across colonies and countries, depending on availability of researchers, volunteers and nation‐specific surveillance policy. Colonies are sorted by their country of origin and hence the colours on the left correspond to the country colour code in part (a). (c) Within‐colony epidemiology was modelled in terms of the four state variables of susceptibles (S), infected (I), recovered (R) and deceased (D). Different types of data (colony size estimates from AOS counts, carcass counts and recovered birds with dark‐tinted irises) entered at different points of the epidemiological model.

In this paper, we demonstrate the utility of using data from the Northeast Atlantic distribution of a single sentinel species, the Northern Gannet (*Morus bassanus*—Figure 1a) to model infection dynamics. Gannets were one of the most severely affected seabird species during an outbreak of HPAI H5N1 clade 2.3.4.4b in 2022 and were impacted across their global distribution (Lane et al., 2024). We use opportunistic carcass counts from all Northeast Atlantic Gannet colonies (Figure 1b) to fit an epidemiological model (Figure 1c) that simultaneously captures three types of transmission: (1) Within‐colony transmission estimating basic epidemiological parameters (such as $R_{0}$) to quantify contagiousness and detect any evidence for heterogeneities in local transmission. (2) Among‐colony transmission estimating metapopulation connectivity in transmission, to infer the rate of exchange of infections between colonies at different distances. (3). Extra‐network transmission using the metapopulation as a sentinel network, effectively, a system of observation nodes, so that any residual unexplained, spatial patterns of HPAI outbreaks were used as a proxy for external incursions originating from the broader seabird community.

Crucially, these epidemiological patterns are non‐independent. Therefore, within‐colony, among‐colony and extra‐network transmission must be quantified in tandem, while also accounting for observation biases. Our novel modelling framework addresses these challenges using imperfect field observations, helping to underpin One Health approaches to wildlife threats and livestock or human spillovers.

## 2 METHODS

### 2.1 Data collection

The locations of 46 colonies extant in 2022 form the colony network were described in detail in Jeglinski et al. (2023). We combined the Icelandic colonies of the Vestmannaeyjar (Brandur, Geldungur, Hellisey and Sulnasker) into one Westman Islands colony complex (Figure 1a). Biologically realistic connectivity between colonies is best based on across‐the‐sea rather than Euclidean distances which we derived using the R package gdistance (Van Etten, 2017).

Carcasses were only partially observed because observation effort varied by colony and time in the year, and many birds died where they could not be seen. The carcass data compilation is described in detail in Lane et al. (2024). We associated carcass sightings, counts excluding chicks, with the nearest gannet colonies if dead birds were sighted within 50 km of the colony. Almost all carcasses tested positive to HPAI during spot‐checks conducted by monitoring authorities in our collaborator network. We summarised the resulting data set to weekly carcass numbers for each colony in the gannet colony network (See map in Lane et al., 2024).

For colonies with observation effort, we assumed that the disease was not present 5 weeks before the first case was detected since field workers were present during these weeks at some of these colonies and recorded no unusual mortality. Therefore, even if there was no HPAI effort specifically on these early weeks we set the observed number of carcasses to zero. All other weeks (i.e. the 5 weeks before any detection, and any other, later weeks with, or without effort) were given an NA value, allowing the model to impute cases. Colonies that were never visited were given NA values throughout, requiring complete imputation. The carcass data availability is summarised in Figure 1b.

We used an independent estimate of ~55% total adult survival on Bass Rock (Lane et al., 2024) as auxiliary information for that colony (via an additional component to the model's likelihood, based on the final accumulation of modelled weekly deaths). As updated estimates are produced from mark‐recapture models (Lane et al., 2026), it will become possible to update this for Bass Rock as well as other colonies. Additional indirect evidence of recovery rates came from the finding that surviving birds acquire varying degrees of dark pigmentation in their typically pale irises, hereafter referred to as ‘black‐eyed’ birds (Lane et al., 2024). This diagnostic, collected for a sample of birds at Bass Rock (*n* = 87) and Grassholm (*n* = 8), was used as a contributor to the joint likelihood of the integrated metapopulation model. We modelled the number of black‐eyed birds as a Binomial variate with number of trials equal to the number (*n*) of birds examined in each colony, and a probability of ‘black‐eye phenotype’ determined by our model as the proportion of recovered birds in these colonies at the end of the outbreak. The assumption that all recovered birds present black eyes will require further field validation.

Initial (pre‐outbreak) gannet colony sizes were provided from the mechanistic Bayesian metapopulation model of the Northeast Atlantic Gannet metapopulation (Jeglinski et al., 2023). We used the posterior median colony size estimates from that model, expressed as apparently occupied sites (AOS).

### 2.2 The epidemiological process model

Opportunities for intra‐specific HPAI transmission occur at different spatial scales. Within a breeding colony, where hundreds, thousands or tens of thousands of pairs interact closely (~2 pairs per *m* ^2^), behaviours such as nest defence, defecation, regurgitation, scavenging and kleptoparasitism (Gorta et al., 2024) all provide potential pathways for disease spread. Adults forage at sea where they interact with conspecifics from the same colony (Langley et al., 2024), and less so with those from other colonies (Wakefield et al., 2013) although they are liable to expand their foraging ranges when exposed (Careen et al., 2024; Jeglinski, Lane, et al., 2024). Immatures form a significant proportion of gannet populations and their more diffuse foraging range and exploratory movements (Votier et al., 2017) increase conspecific encounter probability. There are also several potential disease transmission routes via inter‐specific interactions. Most gannets breed in close proximity to a range of other seabird species including gulls (*Laridae*), skuas (*Stercoraridae*) and auks (*Alcidae*), some of which, such as the great skua (*Stercorarius skua*) and the sandwitch tern (*Thalasseus sandvicensis*) have also suffered severe losses from HPAI at the same time as gannets (Briand et al., 2025; Camphuysen et al., 2022; Knief et al., 2024).

Our approach was based on a network‐structured Susceptible‐Infected‐Recovered‐Deceased (SIRD) model for within‐colony transmission (Figure 1c) equipped with an empirical connectivity function that quantified the transmission among colonies. The model also contained a spatial synchrony term allowing it to detect and quantify residual patterns of infection external to the gannet colony network (extra‐network transmission). Using Bayesian state‐space modelling techniques, we fitted this model to 44 weeks of partial records of occurrence and incidence of the disease as represented by carcass counts (Figure 1b) across the Northwest coast of Europe. This allowed us to fully reconstruct the unfolding of the outbreak across the Northeast Atlantic, estimate overall mortality to gannets and locate the likely hotspots of external contagion.

The model unfolds in discrete time, over 44 weeks, starting 5 weeks before the first case was detected in the Northeast Atlantic Gannet metapopulation on the 15th April 2022. For the ith out of n=43 colonies our state variables refer to the total population size ($P_{i,t}$), and the numbers of susceptible ($S_{i,t}$), infected ($I_{i,t}$), recovered ($R_{i,t}$) and deceased ($D_{i,t}$) individuals in any given week t. We begin by formulating within‐colony epidemiology and then extend the model to account for network interactions.

For a closed colony of size $P_{i,t}$, a local outbreak may be modelled by a generalised version of the law of mass action, in which the transmission rate is written in terms of the proportions of infected (${\hat{I}}_{i,t}=\frac{I_{i,t}}{P_{i,t}}$) and susceptible (${\hat{S}}_{i,t}=\frac{S_{i,t}}{P_{i,t}}$) individuals

(1) $$\lambda_{i,t}=c_{0}{\hat{I}}_{i,t}{\hat{S}}_{i,t}^{c_{1}}$$

where $c_{0}$ is the baseline transmission rate and $c_{1}$ accounts for possible nonlinear responses to the thinning of susceptibles (Finkenstädt & Grenfell, 2000). There are a number of different mechanisms that could give rise to such nonlinearities but our approach is phenomenological, so it does not necessarily correspond to a single mechanism. Values $c_{1}>1$ will yield concave shapes to the transmission, slowing it down disproportionately, as the outbreak progresses. We used a relatively vague prior ($\Gamma \left(100,100\right)$) centred at 1 for $c_{1}$.

The weekly change in the number of cases is the difference between new infections and the loss due to either recovery (ρ) or death (*m*). For the outbreaks to take off within a single colony, it is necessary that this quantity is positive, at the start of the outbreak

(2) $$c_{0}{\hat{I}}_{i,0}{\hat{S}}_{i,0}^{c_{1}}-{\hat{I}}_{i,0}\mathrm{m\rho}>0$$

Which, for the start of the outbreak when ${\hat{S}}_{i,0}\approx 1$ implies

(3) $$R_{0}=\frac{c_{0}}{\mathrm{m\rho}}>1$$

Here, the ratio $R_{0}$ is an approximation of the basic reproduction number of the disease. The approximation is crude, because $R_{0}$ is calculated in discrete time, with an implicit generation time of 1 week, the model's time unit. Also note that, in this discrete‐time model, recovery (a weekly probability) is applied after mortality (also a weekly probability), to the number of survivors, so their combined effect is mρ and not m+ρ as is customarily the case in SIRD in continuous time, where m and ρ are simultaneous rates. Given that the disease took off in many colonies of gannets, we assumed that the above condition held. On that basis, we set the following scaled and joint priors for the three parameters above:

(4) $$\begin{array}{cc} m & =0.3+0.7m^{\prime }\;m^{\prime }∼\text{Beta}\left(10,10\right)\\ \rho & =0.1+0.8\rho^{\prime }\;\rho^{\prime }∼\text{Beta}\left(10,10\right)\\ c_{0} & =\mathrm{mr}\left(1+{c_{0}}^{\prime }\right)\;{c_{0}}^{\prime }∼\Gamma \left(1,10\right) \end{array}$$

The unscaled Beta templates above (primed symbols) are bell‐shaped curves centred at the range's midpoint, but with non‐zero densities at the extremes. The unscaled Gamma template has its mode at zero, implying as a null hypothesis that the basic reproduction number is at least one (because the disease took hold in individual colonies) but without encouraging it towards greater values. The new number of infections each week was modelled as a gamma‐Poisson process (effectively, a negative binomial) with overdispersion parameter θ

(5) $$\begin{array}{ccc} h_{\mathrm{od}} & \sim & \Gamma \left(\theta ,\theta \right)\\ C_{i,t} & \sim & \text{Poisson}\left(h_{\mathrm{od}}P_{i,t}\lambda_{i,t}\right) \end{array}$$

Large values of the parameter θ correspond to reduced dispersion, such that the process tends to a simple Poisson. To ensure that the model was parsimonious with regard to overdispersion (i.e. that it minimised the stochastic variation it used to explain the data) we specified a shrinkage prior for θ:

(6) $$\begin{array}{c} \theta^{\prime }∼\text{Beta}\left(1,100\right)\\ \theta =5+100\left(1-\theta^{\prime }\right) \end{array}$$

We generalise the above model to the metapopulation, by introducing the *effective number of infections* for each colony. This comprises two external contributions, in addition to within‐colony cases. The first comes from other gannet colonies according to some distance‐based *connectivity* matrix g between colonies.

(7) $$g=\left\{g_{j,i},n\times n\right\}$$

Note that this is not a dispersal process between colonies, since breeding gannets are highly faithful to their breeding colony and nest site. Instead, it represents some other form of transmission between gannets at different colonies; for example, transmission at sea, during foraging, or transmission mediated by non‐breeding gannets that nevertheless associate with colonies enough to contract the disease and die where they can be counted among the colony carcasses (Boulinier, 2023).

Notably, the model uses the number of AOS as an approximation of breeding pairs and therefore a proxy for colony size. So, among‐colony transmission may occur by single individuals that have lost their partner, and therefore, even if themselves alive, are considered lost to the breeding population due to the overall process of mortality.

Although our phenomenological model cannot identify the mechanisms in operation, it does nevertheless allow us to quantify the emergent epidemiological connectivity. Note that this is not the same as dispersal connectivity, such as might be measured via animal telemetry or ringing data because it is also shaped by additional behavioural and physical constraints that may affect contact and transfer (Boulinier, 2023).

In general, $g\left(d_{i,j}\right):{\mathbb{R}}_{0}^{+}\to \left[0,1\right]$ can be any monotonically decreasing function of the at‐sea distance $d_{\mathrm{ij}}$ between colonies. Here, we used an exponential decay, in some decay parameter $\delta_{G}$ (such that, when $\delta_{G}=0$ we have distance‐independent mixing).

(8) $$g_{j,i}=g\left(d_{i,j}\right)=\exp\left(-\delta_{G}d_{ij}\right)$$

The effective number of infections arriving at the *i*th colony is therefore.

(9) $$G_{i,t}=\sum_{j=1}^{n}I_{j,t}g\left(d_{i,j}\right)$$

The transmission (Equation 1) then becomes (for ${\hat{G}}_{i,t}=\frac{G_{i,t}}{P_{i,t}}$)

(10) $$\lambda_{i,t}=c_{0}{\hat{G}}_{i,t}{\hat{S}}_{i,t}^{c_{1}}$$

This model has two distinct advantages. First, it subsumes within‐colony transmission, since it reverts to the single‐colony dynamics for $n=1,d_{j,j}=0$. Second, the interaction between different colonies via the effective number of infected individuals allows us to think about the process as a case of local mixing between resident susceptible individuals ($S_{i}$) and a distance‐discounted number of infected individuals from across the entire metapopulation.

Having accounted for within‐colony and among‐colony (collectively, intra‐network) transmission, any residual patterns in the timing of the disease onset at different colonies can be assigned to extra‐network incursions into the breeding gannet population, for example, from other species, or from non‐breeding gannets that may show no association with the colony network. For instance, consider a gannet colony that presents HPAI at a time when none of the surrounding, infected gannet colonies are close enough to represent a credible transmission route. The more geographically distant such colonies are and the longer the time elapsed since a past infection occurred, the more likely it is that the onset of HPAI in the focal colony was the result of another vector. During statistical inference, the model performs this calculation for all colonies at once, while at the same time accounting for intra‐network transmission. Furthermore, the model can connect the extra‐network residuals for all points in space into a spatial surface of the likelihood of incursion. Thus, we can infer where unobserved disease hotspots might be, based on unexplained patterns of synchrony in the timing of gannet outbreaks in space without having explicit data on other species.

Consequently, we included a *synchrony* term, a novel feature that uses the gannet colony network as a set of observation points. If the onset of outbreaks in previously unaffected, neighbouring colonies coincided (i.e. was synchronous) in a way that was not already explained by network connectivity, then this was attributed to extra‐network transmission. The *synchrony* term, denoted $H_{i,t}$, follows a similar mathematical form to connectivity but with two key differences: First, it operates on the presence/absence ($U_{j,t}$) of the disease in other colonies (not the number of infected) and, second, it is normalised across all the colonies. These two features allow the synchrony term to operate as a kernel smoother yielding the probability that any given colony (or, more generally, any point in space, see output in Figure 5) is exposed to the disease. The parameter $\delta_{H}$ captures the rate of decay of spatial autocorrelation in the kernel smoother.

(11) $$\begin{array}{c} h_{j,i}=h\left(d_{i,j}\right)=\exp\left(-\delta_{H}d_{\mathrm{ij}}\right)\\ H_{i,t}=\frac{\sum_{j=1}^{n}U_{j,t}h\left(d_{i,j}\right)}{\sum_{j=1}^{n}h\left(d_{i,j}\right)} \end{array}$$

In keeping with the rest of the model, the synchrony term needs to contribute to the effective number of infected in a colony, however, it is not clear what the strength of this extra‐network forcing, so it was necessary to introduce an additional parameter ($a_{H}$) to quantify that. The complete transmission equation (Equation 10) then becomes:

(12) $$\lambda_{i,t}=c_{0}\frac{G_{i,t}+a_{H}H_{i,t}}{P_{i,t}}{\hat{S}}_{i,t}^{c_{1}}$$

Demographic stochasticity can operate at any stage of an SIRD model (mortality, recovery, contagion) but it is likely to be more influential when the number of infected is small. Arguably, stochastic events are therefore more important in igniting a colony outbreak, and less so when there are hundreds of cases. Our overdispersed contagion term (refer to Equations 5 and 12) captures stochasticity in within‐colony, among‐colony and between‐species transmission. The update equations were as follows:

(13) $$\begin{array}{c} \begin{array}{ccc} S_{j,t+1} & = & \left(1-M\right)\left(S_{j,t}-C_{j,t}\right)\\ I_{j,t+1} & = & \left(1-m\right)\left(I_{j,t}\left(1-\rho \right)+C_{j,t}\right)\\ R_{j,t+1} & = & \left(1-M\right)\left(R_{j,t}+\rho I_{j,t}\right)\\ D_{j,t+1} & = & M\left(R_{j,t}+\rho I_{j,t}+S_{j,t}-C_{j,t}\right)+m\left(I_{j,t}\left(1-\rho \right)+C_{j,t}\right)\\ C_{i,t} & \sim & \text{Poisson}\left(h_{\mathrm{od}}c_{0}\left(G_{i,t}+a_{H}H_{i,t}\right){\hat{S}}_{i,t}^{c_{1}}\right) \end{array} \end{array}$$

Here, M and m are, respectively, the background and avian flu mortalities, making the plausible assumption that m>>M, and that background mortality of infected birds is subsumed into m. ρ is the weekly probability of recovery of infected individuals and $C_{j,t}$ is the number of new contagions. The JAGS code provided in the Supplements better illustrates the detailed bookkeeping that was required to account for the model's discrete, weekly time scale. We set the probability of background (non HPAI), weekly mortality to 0.1%, based on the order of magnitude implied by published estimates of annual survival during HPAI‐free years (Wanless et al., 2006).

### 2.3 The observation model

Binomial observation processes in MCMC models of this complexity are numerically challenging because, especially at the adaptation stage, the Markov chain can visit parameter values that can yield zero probabilities which can terminate model fitting when the observations are not zero. We therefore modelled the carcass counts via the more forgiving normal approximation of a binomial process. Specifically,

(14) $${\hat{D}}_{j,t}∼N\left(D_{j,t}\epsilon_{i,t}\phi ,\sigma_{i,t}\right)$$

where $\epsilon_{j,t}\in \left\{0,1\right\}$ is an indicator variable taking the value 1 when observation effort was expended on colony i in week t, and ϕ is the probability of carcass detection. To approximate a Binomial detection process with probability $\epsilon_{i,t}\phi$, the standard deviation was set to $\sigma_{i,j}=\sqrt{D_{j,t}\phi \left(1-\phi \right)}$.

For the two colonies (Bass and Grassholm) where we had final outcomes (proportions recovered and likely proportions dead), we formulated subsidiary parts of the likelihood as binomial distributions where the overall probability of having died or having contacted and recovered from the infection was informed by the model's current estimate of overall outcomes.

An estimate of total mortality Df was provided for Bass Rock and this was incorporated via a binomial likelihood component

(15) $$\mathrm{Df}∼B\left(P_{0},\mathrm{df}\right)$$

where df was defined at the proportion of deaths in the Bass Rock population imputed by the model.

An estimate of the number of recoveries $Rf_{+}=\left(24,2\right)$ was provided for Grassholm and the Bass Rock, based on the black‐eye diagnostic, from examining $\mathrm{Rf}=\left(87,8\right)$, and this was incorporated via a binomial likelihood component

(16) $$Rf_{+}∼B\left(\mathrm{Rf},\frac{R_{t\max}}{R_{t\max}+S_{t\max}}\right)$$

Inference was performed on the integrated data set simultaneously for process and observation parameters allowing these small pieces of auxiliary information to corroborate the key parameters of the model indirectly. For instance, if the epidemiological parameters currently being considered by the MCMC chain were unlikely to give overall final proportions of mortality or recovery consistent with the auxiliary observations for these two colonies, then the epidemiological parameters would incur a decremental likelihood penalty.

### 2.4 Model fitting, validation and sensitivity

The above model was implemented in the symbolic language JAGS (Plummer, 2003) and run within the R environment (R Core Team, 2021). The model was adapted for 2000 iterations of the MCMC, burnt‐in for 80,000 iterations and 8000 iterations were retained post‐burn‐in as a sample from the joint posterior. Four chains, run in parallel to evaluate mixing and convergence take 6 days on a multicore AMD 2.9 Ghz Ryzen.

The model was validated by simulating data from the same process as the one implemented in model fitting with known parameter values, and then fitting the model to these data, to retrieve the known parameter values. Prior sensitivity was addressed by relaxing parameter priors around their means to the maximum point that would still permit model convergence. Model convergence was evaluated by visual inspection of four simultaneous chains and via the psrf Gelman‐Rubin diagnostic (Gelman & Rubin, 1992).

We further investigated the sensitivity of our results to two key assumptions in the model. To facilitate comparisons, we switched off particular features of the entire model with the help of relevant parameters and refitted to data. First, we examined the model's output under the assumption of colony‐level perfect mixing. We aimed to understand how different the colony‐scale epidemiology inferences would be without nonlinear transmission by setting the transmission exponent $c_{1}$ to 1, and refitting the model to the data. Second, we explored the model's output under the assumption of a gannet‐only model. Here, we aimed to quantify whether the among‐colony, gannet‐only transmission ranges and overall rates would change in response to this constraint. We did this by setting the synchrony parameter $a_{H}$ to zero.

## 3 RESULTS

### 3.1 Within‐colony transmission

The within‐colony part of the model estimated four key epidemiological parameters pertaining to weekly mortality (m), recovery (ρ), the basic reproduction number ($R_{0}$) and within‐colony contact mixing ($c_{1}$) (see also prior‐posterior plots in Figure S2).

Individuals that contracted the disease, died with a weekly probability *m* = 0.7 (95% CI: 0.64–0.76; Figure S2g). The survivors recovered with a weekly probability *ρ* = 0.85 (95% CI: 0.81–0.88; Figure S2h). We used these median values on a hypothetical cohort of 1000 individuals assumed to be infected on the same week to calculate total recoveries $R_{\mathrm{tot}}$, by iterating until all individuals were either dead or recovered.

(17) $$\begin{array}{cc} I_{t+1} & =\left(1-m\right)\left(1-\rho \right)I_{t}\\ R_{\mathrm{tot}} & =\sum_{t=0}^{\infty }\left(1-m\right)\rho I_{t} \end{array}$$

We calculate that, of such a cohort of infected individuals, only 26% would be expected to survive.

*R*_0_, the number of new cases generated by a single incoming case to an uninfected population is used in epidemiology as a measure of the initial force of infection and hence its chances of leading to full outbreaks. Our estimate, *R*_0_ = 2.03 (95% CI: 1.84–2.33) is consistent with the range of 1.2–5.55 reported in the literature for within‐flock transmission in poultry (Kirkeby & Ward, 2022).

The model's contact mixing parameter ($c_{1}$) detects and quantifies deviations from perfect mixing. Our model estimated it to be clearly greater than 1 (median *c*_1_ = 1.41, 95% CI: 1.18–1.65, Figure S2b), providing strong evidence of a decelerating transmission process during a local outbreak, a pattern observed in poultry (Kim & Cho, 2021; Ward et al., 2009) but thus far only hypothesised for wild birds (Haman et al., 2024).

**FIGURE 2:**
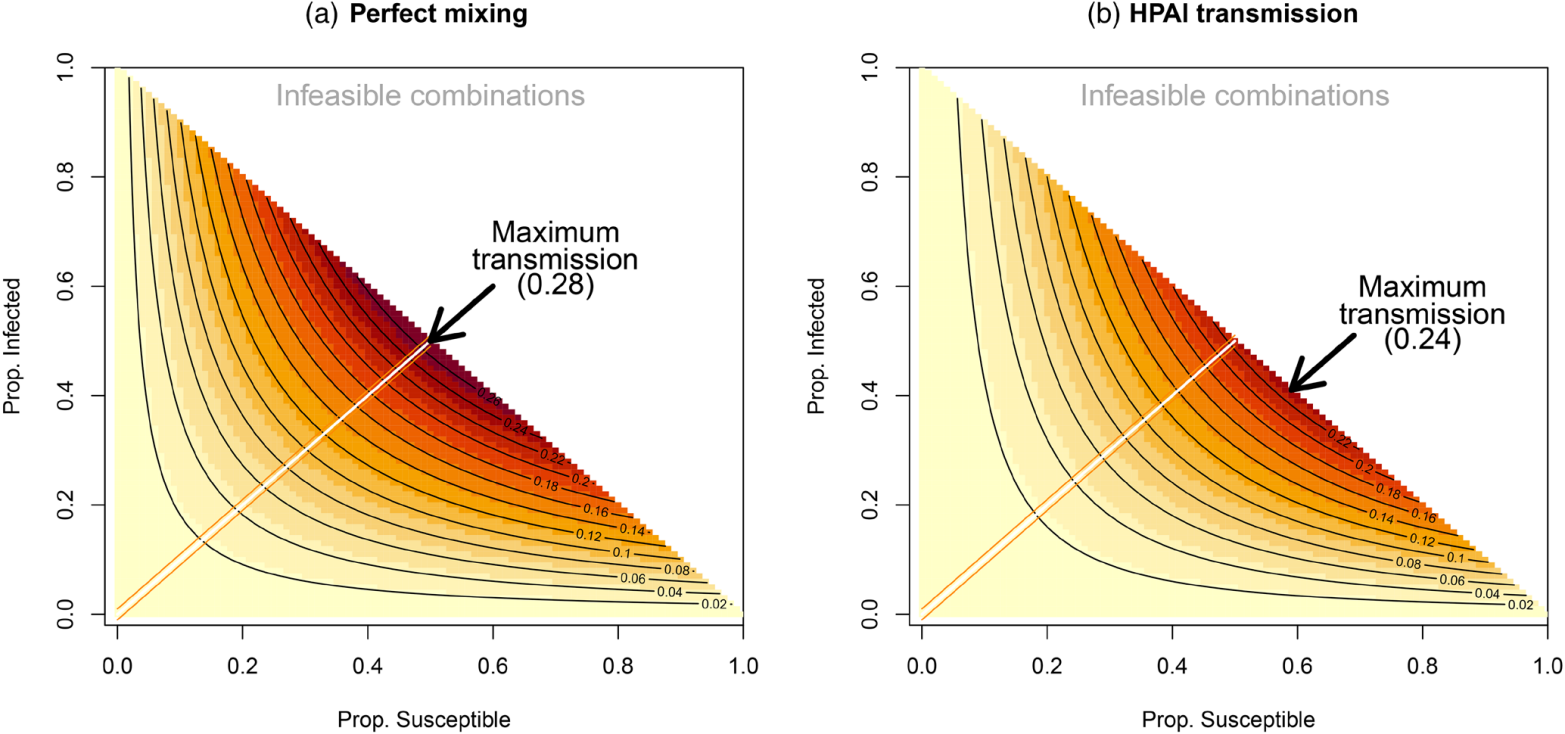
The predicted rate of disease transmission within a colony, for different combinations of Infected and Susceptible proportions, under perfect mixing (a) compared to the transmission rates estimated by the model for HPAI (b). The values of transmission shown as different colours are derived from Equation (1), based on the estimated (median) values of $c_{0}$ and $c_{1}$ (the two contact mixing parameters). Combinations of susceptible (S) and infected (I) proportions adding to more than 1 are infeasible. Combinations adding to less than 1 are possible because there may also be a proportion of recovered individuals.

We explored the importance of this result in comparison to a model that was specified for perfect mixing (i.e. $c_{1}=1$). The comparison (Figure 2) indicates that the maximum rate of transmission (λ, see Equation 10) is likely to be lower than perfect mixing and is likely to occur earlier in the outbreak (when the proportion of infected is lower than the proportion of susceptibles). The parameters for both plots in Figure 2 were derived from fitting the entire model to the full data set under the two assumptions, so as to control for any other confounding effects.

The model accounted for the fact that according to field workers, detectable carcasses could remain on‐site for up to 4 weeks, and the key detectability parameter of the observation model (Figure S2i) indicated that of all the deaths, only 9% (95% CI: 8.5%–9.3%) generated detectable carcasses. We assumed that undetected birds may have died at sea, away from the colony, but we also made the plausible assumption that most dead birds detected near a colony were associated with that colony as breeders. This low level of carcass detection is compounded by the fact that not all colonies were visited during the outbreak and that observed colonies were not visited every week (Figure 1b).

### 3.2 Among‐colony transmission

**FIGURE 3:**
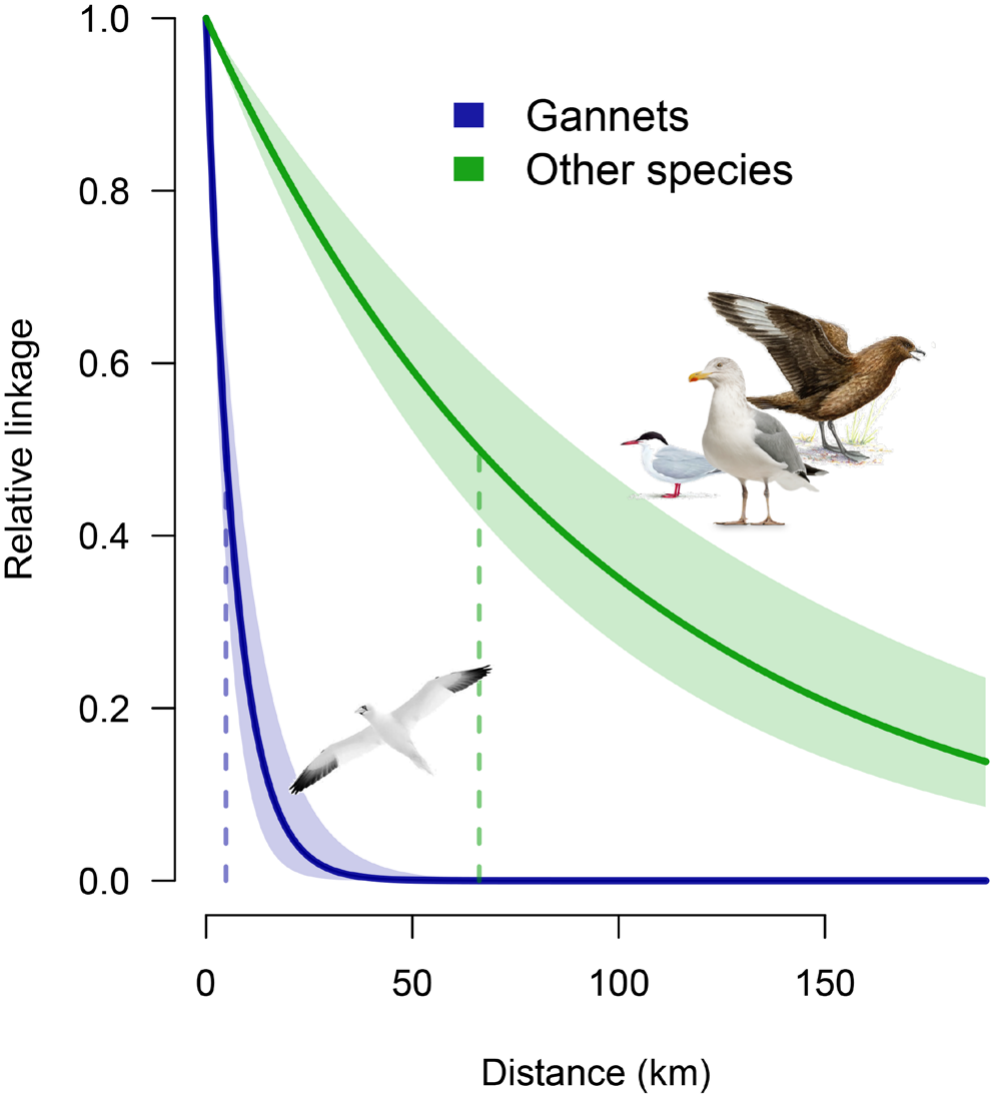
A comparison of the estimated spatial scales of connectivity and synchrony. The connectivity term in the model accounts for new infections occurring as a result of interactions between gannet colonies, whereas the synchrony term quantifies incursions that are coming from outside the breeding network. These are purely empirical functions in the model and postulate no particular mechanism for these transmission dynamics. A measure of the scale over which each process operates is given by the half‐distances of each curve (pointed out by the dashed lines). The shaded areas correspond to the 95% posterior credible intervals. The interpretation of the *y*‐axis (labelled ‘relative linkage’) is different for the two curves. For connectivity, it represents the effective presence, in a given focal colony, of an individual breeding at another colony. The maximum of 1 applies to an infected individual that breeds at the focal colony (hence, inter‐colony distance of zero). For synchrony, the *y*‐axis represents the level of autocorrelation in the residual patterns of transmission (i.e. the rate of influx of non‐gannet cases). A colony is perfectly autocorrelated with its own dynamics, hence the value 1, at distance zero.

With asymptotically decaying processes such as connectivity, it is not possible to define a strict maximum range (i.e. there is always a small probability that the disease will be exchanged between two colonies, even if they are very distant). Hence, to allow us to discuss the spatial scale of connectivity, we defined the *half‐distance of connectivity* as the distance at which an infected individual from another colony contributes half of itself to the effective number of infecteds in a focal colony, compared to a resident breeder. We found that the spatial scale of transmission among colonies was limited (Figure 3), the half‐distance of connectivity (blue dashed line in Figure 3) was estimated as 5 km, a small distance compared to the median distance between nearest‐neighbour colonies of 94 km (range 9–542 km).

### 3.3 Extra‐network transmission

In a similar way to connectivity, we defined the *half‐distance of synchrony* as the distance at which correlation in the rate of external influx of non‐gannet cases between two colonies drops to 0.5 (assuming a perfect autocorrelation of 1, at zero distance). A comparison of spatial scales between transmission processes across the network (Figure 3) indicated that mechanisms of among‐colony transmission (blue dashed line in Figure 3) operate at considerably smaller scales (connectivity half‐distance of 5 km) compared to the extra‐network transmission mechanisms (synchrony half‐distance of 66 km—shown by green dashed line in Figure 3).

Refitting a version of the model to the data without the synchrony term led to two changes. First, the connectivity parameter $\delta_{G}$ decreased in value from 1.44 × 10^−4^ (95% CI: 9.6 × 10^−5^–2.11 × 10^−4^) to 3.6 × 10^−5^ (95% CI: 3.1 × 10^−5^–4.4 × 10^−5^). This implied a half‐distance of connectivity equal to 19 km, approximately, a quadrupling of the connectivity range between gannet colonies compared to the model with both synchrony and connectivity. However, at the same time, this connectivity‐only model did not produce outbreaks in some remote colonies, such as Barra head, Fair Isle, Flannan Isles, Les Etacs, Mykinesholmur, Ortac, Rouzic, St Kilda even though in some of them (e.g. Les Etacs, Mykinesholmur, Ortac, Rouzic, St Kilda) there were carcass detections.

### 3.4 Reconstructing the epizootic across time

**FIGURE 4:**
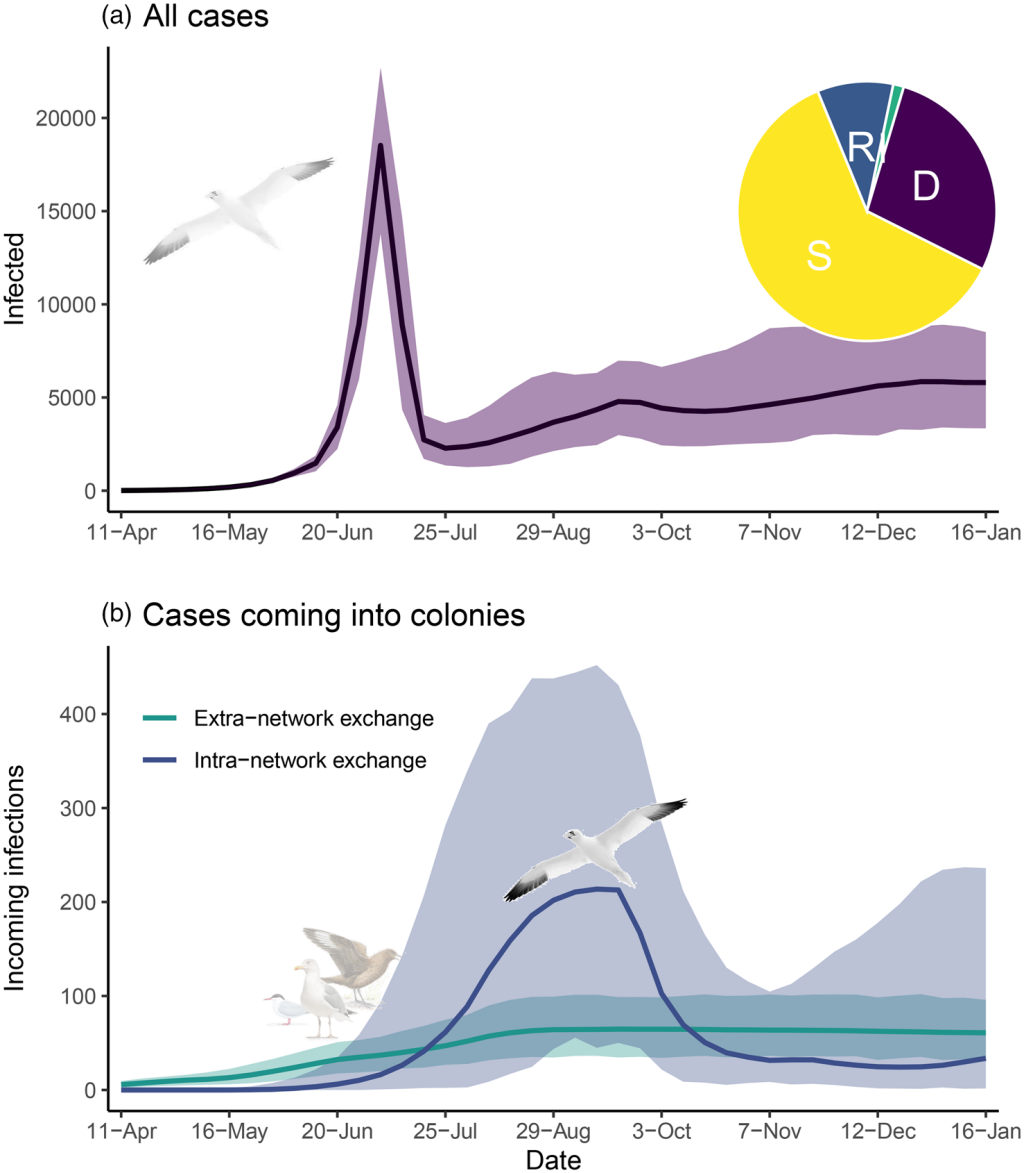
(a) Prevalence across time indicated by median total number of cases (solid curve) and associated 95% credible intervals (purple band). The estimated proportion of animals in each state (S = susceptible, I = infected, R‐recovered, D‐deceased) on week 43 (at the end of the outbreak), is shown as the inset pie chart. (b) The relative strength of incoming infections across all colonies, showing a comparison between intra‐network infections, compared to infections due to external sources.

**FIGURE 5:**
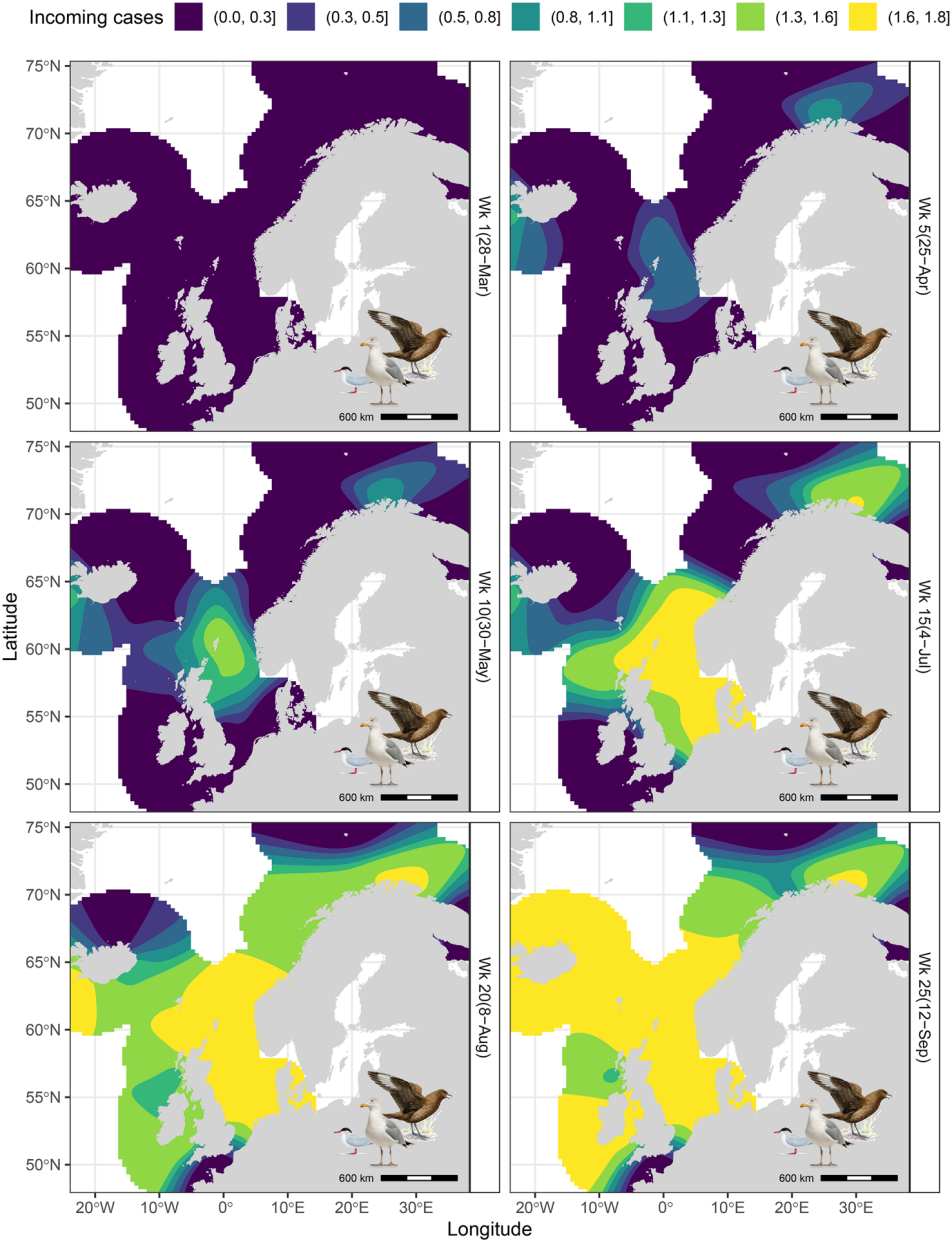
The reconstructed prevalence of the extra‐network distribution of disease as inferred by the synchrony term of the model. This is indicative of the spread of the disease in the broader host community, excluding gannets. Prediction cut‐off distance was determined by setting a small‐value (0.01) cut‐off to the aggregate kernel weights.

The aggregate number of infections across the metapopulation comprised both endemic cases to each colony as well as imported cases from other colonies, or caused by other species. The overall number of cases across Europe appears to have peaked in early July 2022 (Figure 4a) at which time we estimate that there were a median 1.85 × 10^4^ infected gannets (95% CI: 1.38 × 10^4^–2.27 × 10^4^). A direct comparison of colony‐level expected counts with observed carcass counts can be seen in example reconstructions of carcass time series in Figure 5, in the Supplements. The median fatalities at the end of the outbreak corresponded to 28% of the population (95% CI: 21–34%).

We explored the relative contributions of incoming cases, calculated as coming from within, versus outside the gannet breeding network. We added together the number of cases of each type across colonies to arrive at two time series of incoming cases (Figure 4b). These indicated that incoming gannet cases were initially lower than the extra‐network cases. Indeed, the exchange of infected gannets between the network (the blue curve in Figure 4b) only peaked 2 months after the peak in overall cases (Figure 4a). Hence, early outbreaks in many colonies were more likely to have been started by infected individuals from other species (extra‐network transmission—the green curve in Figure 4b). Although there was considerable uncertainty in quantifying these signals, intra‐network transmission took over as the main contributor of imported cases in the end of July 2022, and remained so until October 2022, against a constant backdrop of low‐level extra‐network incursions. The strength and effect of the HPAI outbreak varied across the gannet colony network (Table S1 in the Supporting Information).

### 3.5 Spatiotemporal reconstruction of HPAI spread

Having separated the contribution of connectivity and synchrony mechanisms, we can illustrate the spatiotemporal effects of the synchrony mechanism while controlling for the intra‐network dynamics of the disease. We can therefore pose the question: what is the force of infection that would be experienced at any point in space, as a result of the contagion status in the broader host community? We used our fitted model parameters on extra‐network transmission to answer this question, hence generating a reconstruction of the 2022 unfolding of HPAI beyond the gannet network. As is the case with any point‐transect observation system in wildlife (Buckland et al., 2001) and disease (Nelli et al., 2020), these insights can only extend up to some effective detection distance from the observation network, and therefore our network can only detect external disease hot spots in its vicinity. Here, we used the criterion that no predictions would be attempted for points in space whose synchrony from the nearest gannet colony was less than 1%, which for the parameter estimates of $\delta_{H}$ in our model corresponded to 440 km.

The time sequence of six maps in Figure 5 shows a combination of distant foci and localised travelling waves. The disease was first detected in gannets, in Iceland (Lane et al., 2024), but according to our model there were already two additional foci of infection in Northern Scotland and Northern Norway. These persisted in a segregated way until after week 15 (July 2022), at which time there appeared to be sufficient connection between the Icelandic and Scottish parts. The Norwegian focus never reached the level of the rest of the Northeast Atlantic map and neither did it become as connected as the remainder of the map. Despite these fragmentary patterns, the maps also show discernible patterns of spatially aggregated prevalence (Figures 4b and 5) indicating that although the epizootic was triggered at more than one simultaneous focus, the spread of the disease within the broader multispecies community is nevertheless spatially coherent and predictable.

## 4 DISCUSSION

We have used intermittent and partial counts of carcases from a severely affected species, the Northern gannet, to reconstruct the temporal and spatial pattern of HPAI spread during the 2022 Northeast Atlantic epizootic. Our findings reveal the spatial extent of the outbreak, not just in the focal species (Figure 4), but by‐proxy, in the affected seabird community (Figure 5). Our novel approach accounted for within‐colony, between colony, and extra‐network transmission, demonstrating how to use routinely collected, but currently under‐utilised carcass data for dynamic disease surveillance and spatial prioritisation. Our epidemiological parameter estimates suggest that the 2022 strains of HPAI were highly virulent and transmissible over wide geographical ranges, within the gannet network, and beyond. Our overall mortality estimate of ~30% of breeders agrees with previous, colony‐specific studies (Grémillet et al., 2023; Lane et al., 2024), but is derived independently at a scale exceeding the extent of any previous study.

We have determined that transmission, within single colonies, is heterogeneous. Our parameter estimates, with and without perfect mixing provide evidence for curvature in the within‐colony transmission process (Figure 2) that may lead to earlier and lower peaks in the time series of cases, compared to a homogeneous contagion process. Within the broader epidemiological literature (Gomes et al., 2022), such curvature is likely to be caused by heterogeneities in the behaviour or immune resistance of animals, or in their spatial arrangement. For instance, our finding might point to the existence of early‐phase super‐spreaders with higher‐than‐average mobility in the colony (e.g. pre‐breeders or non‐breeders unconstrained by nest‐site fidelity) and are therefore likely to contract the disease early, spread it wide, but also likely to die out early during the outbreak. The curvature might also be the result of fine‐scale spatial or topographical structuring in the colony. For instance, in high density spatial arrangements, early infected residents may quickly pass the disease to their neighbours in topographically similar terrain (e.g. horizontal plains) but transmission may slow down as topographical heterogeneity reduces direct contact between neighbouring nests (e.g. on vertical cliffs) or after first deaths have thinned the density on the breeding colony. Although our estimate of the contact mixing parameter ($c_{1}$) cannot identify the mechanism underlying heterogeneity, the diverse epidemiological outcomes estimated for different colonies via our approach (see Table S1 and Figure S1 in the Supporting Information) may indicate that differences are between colonies (e.g. topography) rather than individuals (e.g. super‐spreaders). Additional data, for instance, observational, experimental or behavioural data such as regurgitation or territorial defence, as well as a mapping of how location occupancy develops during the breeding season as birds arrive or leave, especially during periods of high mortality, could help elucidate these ambiguities. Geomorphological and disturbance data could also be used as covariates in explaining colony‐level differences in contagion patterns.

Our results pertaining to among‐colony connectivity indicate that direct transmission between gannet colonies is probably a rare event, given the very short connectivity half‐distances involved (Figure 3). The connectivity term in the model is phenomenological, relying on matching the patterns in the outbreaks of individual colonies to their being surrounded by proximate colonies with high HPAI burdens. Such a correlational approach cannot uniquely identify transmission contacts between individual gannets on land or at sea. This would require detailed data on HPAI strains and would rely on being able to discriminate and reconstruct inter‐colony incursions (Andrew et al., 2024; Caliendo et al., 2022). The implicit, and untested, assumption in such an approach would be that viral evolution is faster than the rate of spread of the disease in the colony network.

The finding that the main disease outbreak was initially driven by extra‐network transmission (Figure 4b) indicates that other disease vectors may have functioned as conduits for HPAI. These vectors could be other seabird species that breed in spatial proximity or forage in overlapping ranges. For instance, skuas frequently interact with gannets and were also greatly impacted at the same time and, in Scotland, by the same HPAI strain as gannets (Briand et al., 2025). Alternatively, disease may have been carried to adults by immature prospecting gannets, that move over larger spatial scales than breeders (Votier et al., 2011), adult non‐breeders (sabbaticals) (Votier et al., 2017), or gannets that lost their chick or partner in the outbreak and performed unusual long‐distance movements (Jeglinski, Lane, et al., 2024). Differentiating between these two mechanisms is challenging, however. The carcass observations do not distinguish between breeders and non‐breeders, so probably consist of breeders and colony‐visiting non‐breeders that died there, thus inflating the carcass counts. The intra‐network connectivity term of the model is designed to capture infection fluxes that are consistent with the colony locations and sizes of the breeding gannet network, but non‐breeders, whether immature or adult, are also likely to move in association with the metapopulation network. In contrast, the locations and sizes of colonies of the collective host community, from where external incursions would come, do not exactly coincide with the gannet network. The phenomenological terms for connectivity and synchrony refer to latent (unobserved), stochastic processes and therefore their partitioning of effects between conspecifics and heterospecifics will not be perfect. Some immature gannets may keep no association with breeding colonies and therefore contribute to the synchrony term and, conversely, where different species breed in similar numbers and locations as gannets, they may contribute to the connectivity term. Despite these imperfections, however, we suggest that the extra‐network curve captured by our model, at the level of the whole European breeding metapopulation, predominantly lends support to the idea that HPAI is triggered by between‐species transmission. Our suggestion is supported by the fact that refitting the model without an extra‐network (synchrony) term did not produce outbreaks for several colonies where cases were in fact observed and where the full model (with synchrony) yielded local pulses in prevalence.

The range of prevalence values used for plotting Figures 4 and 5 differ. We defined prevalence as the total number of cases estimated by the model for any given week at any given location. Figure 4a shows all cases generated by intra‐and inter‐colony and extra‐network transmission. At their peak, cases go up to 20,000, which includes all cases generated by intra‐colony transmission. In contrast Figure 4b shows only inter‐colony transmission (median peaks at around 200 for the whole network), or cases estimated to be coming from outside the gannet network of breeders (median peaks around 80 for the whole network). Maximum prevalence values in Figure 4a are much larger than numbers in Figure 4b because the main contributor of new cases is intra‐colony transmission, not the long‐distance exchange of individuals between the colonies or outside the network. Maximum prevalence values in Figure 5 merely reach 1.8 cases per unit area, because these estimates are spread across space, rather than aggregated. However, these differences in maximum prevalence between contributing mechanisms should not be used as a measure for their importance in spreading the disease, since a single case created over a long distance can have a much larger long‐term impact (e.g. by seeding an outbreak in a previously unaffected colony) than many contagions created locally that remain there.

The wide variation in final outcomes between different colonies is interesting, since our transmission model did not employ covariates that could distinguish between known or unknown colony characteristics, such as topography. It may be postulated that the inferred variations in the final outcomes between different colonies are the result of among‐colony distances. However, this seems unlikely, since Figure 4 shows that intra‐network connectivity only played an important role later in the outbreak. Given that all‐but‐one colonies contracted HPAI (the small colony of Bjornoya was spared) and that the virulence was estimated to be high, we would have expected greater similarity in final outcomes between colonies. The model therefore appears to have used overdispersion in the transmission term to recreate many of the otherwise unexplained between‐colony mortality outcomes. It is possible that these heterogeneities are signature results of variable cross‐immunity between avian flu viruses in each colony (Tarasiuk et al., 2022), or that the same colony‐specific features that may influence transmission, may also influence our ability to detect and retrieve carcasses. Further field data and analysis will be needed to elucidate the colony‐specific covariates that may modulate the force of infection or bias carcass observation probabilities.

The combination of mass mortalities with the potential detrimental effects of exposure on survivor condition and breeding success (Careen et al., 2024; Lewis et al., 2026) will have unknown population viability consequences for different wild bird species, many of which are already subjected to acute anthropogenic pressures (Runstadler, 2024). Our estimates of global and localised mortality can readily be incorporated into population viability analyses, combined with other threats such as climate change (Jeglinski, Niven, et al., 2024) and used to evaluate the long‐term impacts of HPAI on wild populations (Jeglinski et al., in prep.).

The recent scale and severity of the outbreak caused by subtype H5N1 clade 2.3.4.4b HPAIV (Krammer et al., 2025) has surpassed previous epizootics in both spatial (Sacristán et al., 2024) and taxonomic extent (Puryear & Runstadler, 2024). Human risk was until recently classed as low because there had not yet been a mechanism of mammal‐to‐mammal transmission (European Food Safety Authority (EFSA) et al., 2024; WHO, 2024), however evidence is emerging to the contrary (Pardo‐Roa et al., 2025) and cases have increased to 20–140 annually since 2005 (Perez‐Acle et al., 2024), varying from asymptomatic to fatal in outcome. Therefore, the unprecedented nature of H5N1 clade 2.3.4.4b HPAIV necessitates an urgent global effort to understand the ongoing epidemiology and implications of AIV in wild hosts.

Outbreaks in wildlife populations are difficult to mitigate. Interventions such as vaccinations and culls are simply not options for wide‐ranging, threatened animals breeding in colonies of several tens of thousands of individuals. Instead, we are restricted to indirect, potentially disruptive and inefficient interventions (e.g. carcass removal to prevent spread through scavenging (Gold et al., 2026; NatureScot, 2023)) and responsive livestock lockdowns. These interventions either happen in a spatially comprehensive (and extremely costly) form, or, when selectively applied, they are often too little, too late. It is therefore both a pressing and challenging problem in wildlife epidemiology to develop surveillance networks, informing methods of accurate and preventative spatial prediction based on intermittent, partial and heavily error‐prone data. Routine and responsive monitoring and reporting of die‐offs of wild birds from HPAI in most countries are currently underfunded with limited cross‐border coordination. Hence, fortifying monitoring networks is recognised as a top priority (Runstadler, 2024). Here, we have demonstrated how opportunistic observations can be organised into a synthetic framework.

Although heterogeneities in the carcass detection effort were expressly represented in the data, and the model produced an estimate of detection probability (in the range 0.085–0.093), it was not possible to refine this for every colony individually. Therefore, with an expansive data set such as ours, it is likely that observation biases will vary with region, not just because of different field protocols, but also because of oceanographic and geomorphological differences. Given the opportunistic and error‐prone nature of our carcass data, our investigation relied on spatiotemporal breadth rather than localised replication, but our framework can make use of both designs simultaneously. Although high levels of effort (e.g. daily or weekly visits) cannot be attained across the range of species affected, it would be vital to maintain selected, permanent observation posts (a type of disease ‘weather station’, for example, Camphuysen et al., 2022; Ewing & Bouwhuis, 2025; Haman et al., 2024). Maximising the use of different types of data (e.g. drone counts, observations of black eyes, serology sampling, movement tracking) to enhance surveillance efforts is now key to monitoring future risk of a virus of conservation importance, economic cost and pandemic potential (Boulinier, 2023; Lane et al., 2024; Tyndall et al., 2024). In particular, integration with viral sequencing data (Briand et al., 2025; Clessin et al., 2025) should now be considered a priority for modelling approaches. Our study demonstrates that if analysed holistically, carcass data can provide epidemiological insights but also act as an early warning system for continent‐wide patterns of spread. For pandemic preparedness, it is essential that data‐integrative approaches such as ours are placed in the mainstream toolbox of planetary health monitoring.

## AUTHOR CONTRIBUTIONS

Jason Matthiopoulos conceived the idea, developed the model, carried out statistical analysis and wrote the first draft of the MS. Jana W. E. Jeglinski collated the carcass data and helped with the figures. Jana W. E. Jeglinski, Jude V. Lane and Stephen Votier collected the carcass, black eye and aggregate mortality data pertaining to Bass rock and Grassholm. Sarah Wanless, Jude V. Lane, Jana W. E. Jeglinski and Stephen Votier guided the model development with regard to seabird natural history. Emma Cunningham guided the modelling with regard to avian flu specifics. All authors commented critically during model development and contributed to the writing of the submitted version.

## CONFLICT OF INTEREST STATEMENT

The authors declare no conflict of interests.

## ETHICAL APPROVAL

The study required no ethical approval.

## Supporting information


**Figure S1.** Goodness‐of‐fit of the model for the 43 colonies. Grey dots are carcass counts, incomplete because observation effort was intermittent and partial (due to low sightability). The solid lines show the model's reconstruction of the median number of carcasses (and 95% credible intervals in salmon) that would be expected to be seen on any given week.
**Figure S2.** Prior (black curves) and posterior (brown histograms) distributions for the nine key model parameters.

## Data Availability

The model code, data and Quarto script for this paper can be found on the DRYAD public access repository at DOI: https://doi.org/10.5061/dryad.gxd25482f (Matthiopoulos et al., 2026).
