# Supplementary material for: Between‐species contagion drives HPAI transmission and mass mortality in seabirds
Source: J Anim Ecol. 2026 Jul 6;95(8):1488–503. doi: 10.1111/1365-2656.70305 (PMC13439604; doi:10.1111/1365-2656.70305)
Supplement: Supplementary file 1 [file JANE-95-1488-s001.docx]

Supplements

## 1 JAGS code for model

```
###### Model statement ######
AIModDet<-  "model{

  # Initialisation of states
  for(i in 1:n)
  {
    P[i,1]<-P0[i] # Total population is set to colony size
    S[i,1]<-P0[i]-I[i,1] # Total susceptibles
    I[i,1]~dpois(a0) # Initial cases in each colony
    R[i,1]<-0 # No recoveries to begin with
    D[i,1]<-0 # No deaths to begin with
    U[i,1]<-min(1,I[i,1]) # Disease presence in the colony
  }


  # Between-colony linkage matrices
  for (i in 1:n)
  {
    for (j in 1:n)
    {
      g[i,j]<-exp(-(deltaG*di[i,j])) # Connectivity pairwise
      h[i,j]<-exp(-(deltaH*di[i,j])) # Synchrony pairwise
    }

    H[i]<-sum(h[1:n,i]) #Column sums for synchrony
  }


  ### MAIN LOOP ###
  for(t in 1:(Tmax-1)) # Time loop, in units of weeks
  {

    fG[1:n,t]<- (t(I[1:n,t])%*%g[1:n,1:n]) # Connectivity
    fH[1:n,t]<- (t(U[1:n,t])%*%h[1:n,1:n]) # Sychrony
    cumSt[t]<-t-min(3,t-1) # Start of observation window for current carcasses

    for(i in 1:n) # Colony loop
    {

      Ief[i,t]<- (fG[i,t]+aH*fH[i,t]/H[i])/P[i,t] # Per-capita contacts
      GIef[i,t]<-fG[i,t]-I[i,t] # Connectivity incomers
      HIef[i,t]<-aH*fH[i,t]/H[i] # Synchrony incomers

      hod[i,t]~dgamma(theta,theta) # Transmission process overdispersion

      # Transmission process
      contagionsD[i,t]~dpois(hod[i,t]*(P[i,t]*c0*Ief[i,t]*(S[i,t]/P[i,t])^c1))
      contagions[i,t]<-min(S[i,t], contagionsD[i,t])
      Sr[i,t]<-max(0, S[i,t]-contagions[i,t])

      # Deaths due to avian flu
      deathsAI[i,t]<-aiM*I[i,t]

      # Deaths due to background mortality
      deathsNAS[i,t]<-naM*Sr[i,t]
      deathsNAR[i,t]<-naM*R[i,t]

      # Recoveries of survivors
      recoveries[i,t]<-rrr*I[i,t]*(1-aiM)

      # SIR Update equations
      R[i,t+1]<-max(0,R[i,t]+recoveries[i,t]-deathsNAR[i,t])
      I[i,t+1]<-max(0, I[i,t]+contagions[i,t]-deathsAI[i,t]-recoveries[i,t])
      S[i,t+1]<-max(0,S[i,t]-contagions[i,t]-deathsNAS[i,t])
      U[i,t+1]<-min(I[i,t+1],1) # Is disease present in the colony?

      # Total deaths
      deaths[i,t]<-deathsAI[i,t]+deathsNAS[i,t]+deathsNAR[i,t]
      D[i,t+1]<-D[i,t]+deaths[i,t]

      # Current population size
      P[i,t+1]<-I[i,t]+S[i,t]+R[i,t]

      cumDeaths[i,t]<-sum(deaths[i,cumSt[t]:t]) # Carcasses last for three weeks

      # Observation model for data
      Carcass[i,t]~dnorm(cumDeaths[i,t]*esc,1/(cumDeaths[i,t]*esc*(1-esc)+0.001))
      # Observation model wihtout data to be used for goodness-of-fit plots
      Carcass2[i,t]~dnorm(cumDeaths[i,t]*esc,1/(cumDeaths[i,t]*esc*(1-esc)+0.001))
    }

    # Total cases across metapopulation
    Infected[t]<-sum(I[1:n,t])
    # Total incoming conspecific cases
    GItot[t]<-sum(GIef[1:n,t])
    HItot[t]<-sum(HIef[1:n,t])
  }

  # Numbers of individuals in classes, at end of time series
  EndInfc<-sum(I[1:n,Tmax])
  EndRecv<-sum(R[1:n,Tmax])
  EndDed<-sum(D[1:n,Tmax])
  EndSsc<-sum(S[1:n,Tmax])


  # Individuals in classes, at end of time series, by colony
  for(i in 1:n)
  {
  NInfc[i]<-I[i,Tmax]
  NRecv[i]<-R[i,Tmax]
  NDed[i]<-D[i,Tmax]
  NSsc[i]<-S[i,Tmax]
  }

  # Keeps track of external incoming cases (through synchrony mechanism)
  for(ts in 1:9)
  {
    uEf[1:n,ts]<-HIef[1:n,timestamps[ts]]
  }


  # Likelihood component for observed final mortality
  for(i in 1:nMort) # Final deaths - based on empty nests
  {
    muM[i]<-min(0.9999,max(0.0001,D[morts[i],Tmax]/P0[morts[i]]))
    obsMort[i]~dbinom(muM[i],testMort[i])

  }

  # Likelihood component for observed final recoveries
  for(i in 1:nRec) # Final recoveries - based on black-eye inspection
  {
    muR[i]<-min(0.9999,max(0.0001,R[recs[i],Tmax]/(R[recs[i],Tmax]+S[recs[i],Tmax])))
    obsRec[i]~dbinom(muR[i],testRec[i])

  }


 ### Priors ###
  # Connectivity
  deltaHd~dnorm(-11,1) # Null hypothesis of perfect mixing (very low mean value)
  deltaH<-exp(deltaHd) # Transformation to exp scale

  # Synchrony
  deltaGd~dnorm(-11,1) # Null hypothesis of perfect mixing (very low mean value)
  deltaG<-exp(deltaGd) # Transformation to exp scale
  aH~dgamma(0.1,0.1) # Synchrony coefficient

  # Epidemiology
  thetaDum~dbeta(1,100)
  theta<-5+100*(1-thetaDum)
  a0~dgamma(1,10) # Background case rate for initialisation
  # Basic transmission coefficient, derived from requirement that R0>1
  c0cap~dgamma(1,10)
  c0<-(1+c0cap)*rrr*aiM
  # Contact mixing parameter - null hypothesis c1=1, perfect mixing within colony
  c1~dgamma(100,100)
  # Scaling for beta templates. Large values give more informative priors
  tig<-10
  # Weekly recovery probability of surviving infected
  rd~dbeta(tig,tig)
  rrr<-0.1+0.8*rd
  # Weekly mortality probability
  aiMd~dbeta(tig,tig)
  aiM<-0.3+0.7*aiMd
  naM<-0.0001

  # Detectability
  escd~dbeta(1,10) # Detectability prior
  esc<-0.3*escd # Detectability upper limit of 30%

  #data# Tmax,n,P0, Carcass, di , obsMort, obsRec, nMort, nRec, morts, recs
  #data# testMort,testRec, timestamps

  #monitor#  c0,c1,deltaG,aH,deltaH,aiM,rrr,esc,theta, muM,muR,
  #monitor#  NInfc, NRecv, NDed, NSsc,GItot, HItot
  #monitor#  Carcass2, Infected, deaths, uEf, EndInfc, EndRecv, EndDed, EndSsc

  #inits# aiMd
  #
}"
```

## 2 Fitted trajectories

In Figure 6 we present all colony-specific trajectories fitted to the available carcass data. Since the data offer a distorted view of the underlying (unobserved) cases in the population, the reconstructed trajectories shown here are not cases, but, rather, the number of carcasses that an observer should expect to count if they had visited that colony at that point in time. This index also includes the model’s estimate of detectability of carcasses and the fact that dead birds may remain available for detection for up to 4 weeks, on average. We see that despite the good performance of the model (most observations lie within the model’s 95% credible intervals), there is nevertheless a difficulty in capturing the higher observed values, in the sense that the model’s predicted median count (shown as the solid black lines in Figure S1) are always below the peak carcass observations. The model performs much better with regards to the reconstructed duration of the pulses in the data.

Figure S1: Goodness-of-fit of the model for the 43 colonies. Grey dots are carcass counts, incomplete because observation effort was intermittent and partial (due to low sightability). The solid lines show the model’s reconstruction of the median number of carcasses (and 95% credible intervals in salmon) that would be expected to be seen on any given week.

## 3 Individual colony outcomes

Reconstructed final epidemiological outcomes, by colony. Median mortality and recovery percentages are listed, along with their corresponding posterior credible intervals. Estimated colony size is for 2022, measured in AOS.

| Colony | Size | % Mort. | 95% CI | % Recov. | 95% CI |
| --- | --- | --- | --- | --- | --- |
| Ailsa Craig | 30405 | 17 | (2-41) | 6 | (0-14) |
| Barra Head | 10 | 74 | (68-80) | 26 | (20-31) |
| Bass Rock | 63978 | 44 | (44-44) | 15 | (11-20) |
| Bempton Cliff | 18220 | 36 | (17-53) | 13 | (5-19) |
| Bjornoya | 58 | 0 | (0-0) | 0 | (0-0) |
| Westmann Islands | 8067 | 14 | (3-33) | 5 | (1-12) |
| Buholmene | 500 | 47 | (32-63) | 17 | (10-23) |
| Bull Rock | 7550 | 12 | (5-23) | 4 | (1-9) |
| Clare Island | 369 | 40 | (21-57) | 14 | (7-21) |
| Eldey | 14546 | 8 | (4-13) | 3 | (1-5) |
| Fair Isle | 5408 | 41 | (21-57) | 14 | (7-20) |
| Flannan Isles | 8738 | 38 | (15-54) | 13 | (5-21) |
| Foula | 2381 | 45 | (31-58) | 16 | (10-21) |
| Grassholm | 30587 | 17 | (2-41) | 6 | (1-15) |
| Great Saltee | 7957 | 14 | (11-20) | 5 | (3-7) |
| Helgoland | 1115 | 43 | (32-55) | 15 | (11-20) |
| Hermaness | 43467 | 40 | (19-56) | 13 | (5-20) |
| Ireland’s Eye | 918 | 60 | (52-68) | 21 | (16-26) |
| Kharlov Kola Peninsula | 340 | 43 | (0-62) | 15 | (0-22) |
| Kvitvaer | 35 | 69 | (55-78) | 24 | (18-29) |
| Lambay | 1171 | 50 | (42-60) | 18 | (13-22) |
| Les Etacs | 8449 | 1 | (0-41) | 0 | (0-14) |
| Little Skellig | 30569 | 6 | (3-11) | 2 | (1-4) |
| Mykinesholmur | 2658 | 38 | (12-58) | 13 | (3-20) |
| Noss | 18595 | 40 | (18-58) | 14 | (5-20) |
| Oddskjaeren | 26 | 73 | (66-80) | 26 | (20-31) |
| Ortac | 2570 | 3 | (0-63) | 1 | (0-22) |
| Raudinupur | 939 | 44 | (29-60) | 15 | (9-22) |
| Rouzic | 20294 | 1 | (0-7) | 0 | (0-3) |
| Runde | 4095 | 30 | (12-49) | 10 | (4-18) |
| Scar Rocks | 2352 | 39 | (17-56) | 14 | (5-20) |
| Skoruvikbjarg | 945 | 40 | (20-56) | 14 | (7-21) |
| Skrudur | 6557 | 25 | (11-44) | 9 | (3-15) |
| St Kilda | 54723 | 21 | (4-48) | 7 | (1-17) |
| St Ulvoyholmen | 353 | 50 | (35-64) | 18 | (12-23) |
| St. Foroya | 244 | 54 | (39-68) | 19 | (13-25) |
| Storstappen, Gjesvaer | 1437 | 48 | (32-61) | 17 | (10-22) |
| Sula Sgeir | 13871 | 31 | (7-53) | 11 | (1-18) |
| Sule Skerry | 4695 | 58 | (50-68) | 20 | (16-26) |
| Sule Stack | 4922 | 58 | (49-67) | 20 | (15-25) |
| Syltefjord | 865 | 49 | (36-62) | 17 | (12-23) |
| Troup Head | 7674 | 45 | (33-58) | 16 | (11-21) |
| Westray | 1549 | 46 | (32-60) | 16 | (11-22) |

## 4 Prior-posterior plots

In Figure S2 we present the marginal prior and posterior plots for 8 key parameters of the model.

Figure S2: Prior (black curves) and posterior (brown histograms) distributions for the nine key model parameters.
